# Tracking Phenological Changes over 183 Years in Endemic Species of a Mediterranean Mountain (Sierra Nevada, SE Spain) Using Herbarium Specimens

**DOI:** 10.3390/plants13040522

**Published:** 2024-02-14

**Authors:** Katy V. Rondinel-Mendoza, Juan Lorite, Macarena Marín-Rodulfo, Eva M. Cañadas

**Affiliations:** 1 Departamento de Botánica, Facultad de Ciencias, Universidad de Granada, 18071 Granada, Spain; jlorite@ugr.es (J.L.); macamarin@correo.ugr.es (M.M.-R.); ecanadas@ugr.es (E.M.C.); 2 Interuniversity Institute for Earth System Research, University of Granada, 18071 Granada, Spain

**Keywords:** endemic plant, flowering, fruiting, herbarium sheet, global warming, Mediterranean mountain, phenology

## Abstract

Phenological studies have a crucial role in the global change context. The Mediterranean basin constitutes a key study site since strong climate change impacts are expected, particularly in mountain areas such as Sierra Nevada, where we focus. Specifically, we delve into phenological changes in endemic vascular plants over time by analysing data at three scales: entire massif, altitudinal ranges, and particular species, seeking to contribute to stopping biodiversity loss. For this, we analysed 5262 samples of 2129 herbarium sheets from Sierra Nevada, dated from 1837 to 2019, including reproductive structure, complete collection date, and precise location. We found a generalized advancement in phenology at all scales, and particularly in flowering onset and flowering peak. Thus, plants flower on average 11 days earlier now than before the 1970s. Although similar trends have been confirmed for many territories and species, we address plants that have been studied little in the past regarding biotypes and distribution, and which are relevant for conservation. Thus, we analysed phenological changes in endemic plants, mostly threatened, from a crucial hotspot within the Mediterranean hotspot, which is particularly vulnerable to global warming. Our results highlight the urgency of phenological studies by species and of including ecological interactions and effects on their life cycles.

## 1. Introduction

Phenology (i.e., the study of the timing of recurring biological events in the animal and plant world, the causes of their timing (biotic and abiotic forces), and the interrelations among phases of the same or different species [1]) is an integrative science that has achieved a crucial role in the current context of global change [2]. In fact, changes in the timing of phenological events are among the most important indicators of global warming [3,4,5]. Thus, many studies have confirmed that plants are modifying the timing of the development and the shape of their vegetative and reproductive structures in response to global warming [6,7,8]. This could explain the shift in the distribution range of certain species reported as new at the regional or country level [7], or promote speciation [9]. In particular, phenological events at temperate latitudes have advanced by between 1.5 and 2.5 days per decade since the 1970s [7,10]. 

Phenological changes in plants have consequences not only on the reproductive success of the species, but also on cascades at different levels and across functional groups within communities, including decomposers, detritivores, herbivores, predators, pollinators, and seed dispersers [11,12,13]. In this sense, phenological changes may influence the synchronization between flowering and pollinator activity or between fruiting and seed disperser activity, and, thus, the connectivity and gene flow through pollen and seed movements across landscapes [2]. Therefore, plant phenology is extremely relevant for ecological processes and for biodiversity conservation over time, and ultimately for the maintenance of essential ecosystem services [14,15]. Consequently, phenological changes will have significant impacts on agriculture, forestry, human health, and the global economy [16]. 

The Mediterranean basin constitutes a key area for the study of phenological changes, since it is considered one of the regional foci where climate change will exert particularly strong effects [17,18]. Although there have been few previous studies in this region on long-term phenological changes, the limited precedents suggest that global warming and increasing drought frequency have led to major shifts in the timing of phenophases in Mediterranean ecosystems [19]. In particular, [20] found that an increase of 1.4 °C from 1952 to 2000 led to a generalized phenological advancement in recent decades (i.e., on average, leaves unfold 16 days earlier, leaves fall 13 days later, and flowering occurs 6 days earlier).

Within the Mediterranean, mountain areas are especially vulnerable to climate change [21]. This is particularly concerning in summit areas, as they often constitute biodiversity nano-hotspots rich in endemic species [22,23]. Studies of recent changes in vascular plant richness across Europe’s major mountain ranges found that, on average, species moved upslope, and the loss of endemic species was particularly severe in the Mediterranean mountains. However, mountaintop endemic species are unable to adopt vertical migration strategies [24], which is crucial to coping with climate change [25]. Yet, to our knowledge, phenology and phenological changes in plants endemic to the Mediterranean mountains have been poorly addressed and encompass few species (e.g., [26,27]).

One of the main difficulties in studying phenological changes is the limited availability of long-term series data. In this regard, herbarium sheets are a powerful source of spatially and temporally extensive data on plant functional traits, and therefore are very valuable for the study of phenological changes over time [28,29]. Thus, many studies based on herbarium data have revealed changes in reproductive phenology in response to global warming and altered precipitation patterns [30,31,32]. In line with other phenological studies, analyses of herbarium samples have confirmed an advancement in phenology in recent decades [33,34,35]. 

This study presents a comprehensive analysis of the phenological shifts in endemic plants of Sierra Nevada, a Mediterranean mountain massif in southwestern Europe, using herbarium samples from the last 183 years. Sierra Nevada stands out as one of the main biodiversity hotspots within the larger Mediterranean hotspot [22,36], yet is severely threatened by the impacts of climate change [23,37,38]. Remarkably, Sierra Nevada houses the highest peak (Mulhacén, 3482 m asl) in the Iberian Peninsula and supports a high level of plant biodiversity [39]. Its unique ecological setting has attracted numerous renowned botanists since the 19th century [40], resulting in the preservation of a substantial number of herbarium sheets coming from their field expeditions. Consequently, Sierra Nevada serves as an invaluable natural laboratory for investigating phenological changes based on historical herbarium specimens. 

Specifically, our analysis focuses on discerning phenological trends in endemic vascular plants over time, examining whether these trends exhibit consistency across different altitudinal ranges (non-alpine vs. alpine zones) and specific species. The ultimate aim is to discern trends that provide valuable insights into how to address the critical challenge of halting biodiversity loss.

## 2. Methods

### 2.1. Study Area

Sierra Nevada is a small (ca. 2100 km^2^) and isolated mountain range in southeastern Spain (from 36°50′24″ to 37°15′0″ N latitude and 3°44′24″ to 2°35′24″ W longitude) exhibiting a diverse topography and an extensive altitudinal gradient from 200 to 3479 m asl. This geographical uniqueness makes it resemble a sort of continental island, also being the only true alpine region located between the North African mountains (High and Middle Atlas) and the Pyrenees, both several hundreds of kilometres away. 

The climate is a typically Mediterranean mountain type, characterized by cold winters and hot summers with pronounced droughts (July–August). Precipitation, mainly in the winter, ranges from 350 to 1200 mm per year, depending mostly on altitude, and 75% occurs in the form of snow above 2000 m asl. The average annual temperature is 12 °C, with strong day–night and winter–summer fluctuations. In the winter, temperatures can drop to −35 °C, and snow can remain for up to 8 (occasionally up to 10) months in the highest areas [38]. 

Regarding geology, the massif is made up of siliceous rock (i.e., micaschists, phyllites, and quartzites) from the Permo-Triassic surrounded by carbonates (limestones and dolomites) from the Middle-Upper Triassic [41]. The different combinations of climatic conditions and rock types favour the presence of a high level of diversity of habitats and species [42]. In relation to the plant diversity, Sierra Nevada represents one of the most relevant hotspots in the western Mediterranean [43,44], with more than 2348 taxa of vascular plants, including 79 endemic and 16 sub-endemic to Sierra Nevada. It also has 362 taxa inhabiting the alpine zone (about 242 km^2^), representing 79% of the endemism of the whole area [39,45].

### 2.2. Phenological Data

Phenological data were obtained by reviewing the herbarium sheets, which encompassed 89 vascular plant species, including 62 endemic and 16 sub-endemic taxa from Sierra Nevada, plus 11 additional taxa which are also relevant for conservation (see Appendix A). We included all endemic and sub-endemic taxa from Sierra Nevada, except those belonging to the *Poaceae* family due to the inherent difficulty of discerning their phenological stage. A total of 5262 sample “observations” from 2129 herbarium sheets were examined from April 2019 to December 2021. These data came from the main herbaria housing material from Sierra Nevada (herbaria acronyms according to [46]): GDA-GDAC (1954 samples), MA (2002 samples), SEV (646 samples), MGC (346 samples), JAEN (130 samples), and HUAL (61 samples). We also included digital samples from

G (CJBG source: https://www.ville-ge.ch/musinfo/bd/cjb/chg/index.php?lang=en (accessed on 1 March 2020); 13 samples) and RECOLNAT (source: https://www.recolnat.org/en/, accessed on 1 March 2020; 110 samples). Notably, the time period for the dataset ranged from 1837 to 2019 (see Appendix B).

The herbarium sheets finally selected for this study met the following three criteria, which were applied before obtaining the total number of records: (1) At least 50% of the reproductive structures exhibited good preservation; (2) had a complete collection date, including day, month, and year; and (3) had precise geographical information, either in the form of exact coordinates or sufficiently detailed locality descriptions, enabling us to assign precise coordinates (error < 1 km). 

Thus, in this first part of this study, phenological, spatial, and temporal information for each individual sample of herbarium sheet was recorded as follows: (I) Number of reproductive structures (no. of flower buds “NB”, no. of flowers “FL”, and no. of fruits “FR”) was recorded. (II) Phenological phase, based on the highest quantitative representativeness and state of development of reproductive structures: We established 6 categories: (1) flowering onset, “FL_O” (state of flower bud); (2) flowering peak, “FL_P” (anthesis of the flower ready for pollination); (3) flowering late, “FL_L”(beginning of adult flower wilting); (4) fruiting onset, “FR_O” (beginning of embryo formation or immature fruits); (5) fruiting peak, “FR_P” (ripe fruits and seeds production in ripe fruits); and (6) fruiting late, “FR_L” (very ripe fruits, close to dehiscence). (III) Complete dates of sheet collection (day/month/year) were recorded, taking into account the leap years. These dates were converted into days of the year, (i.e., 30 July 1954 corresponds to the 211th day of the year). We named this variable “Julian date (JD)”. (IV) Geographical position was noted (with coordinates and/or precise localities, allowing coordinates to be assigned in a subsequent step). (V) Altitude data were obtained from coordinates of a digital elevation model (https://www.ign.es/wms-inspire/mapa-raster, accessed on 8 March 2022) using QGIS Desktop 3.24.1 (http://www.qgis.org, accessed on 8 March 2022).

### 2.3. Statistical Analyses

In order to explore temporal shifts in phenology from 1837 to 2019 across the 89 species assessed, we fitted generalized linear models (GLMs with family Poisson and link = log) using the Julian date (JD) as the response variable and the collection year of the herbarium sheet and the phenological phase as independent variables (Julian date ~ year * Phenological phase). In addition, we performed lineal models (LMs) for each phase (JD ~ Year) using the complete dataset. Next, we assessed the consistency of phenological trends across different altitudinal ranges. Thus, we divided the dataset into two groups: (1) samples from herbarium sheets collected above 2400 m asl (alpine zone) and (2) samples from sheets collected below 2400 m asl (non-alpine zone). GLMs were fitted for each altitudinal range dataset and LMs were used to explore trends according to phenological phase, as described above. To evaluate the model’s performance, we computed *p*-values and pseudo-R-squared values for all fitted models compared to the null models. For this purpose, the “nagelkerke” function from the “rcompanion” library was used. In the results section, we present the Nagelkerke pseudo R^2^ [47] for GLMs and adjusted R^2^ for LMs. 

In order to assess changes by species, we focused on flowering peaks and fruiting peaks, because we had a greater number of herbarium sheets for these phases. In particular, we used data from those species with at least 5 samples per period (≤1969 vs. ≥1970) for each phase. These conditions were met by 18 taxa for the flowering peak and 12 taxa for the fruiting peak phases. Subsequently, to highlight the number of days of advancement or delay in phenology, we divided the dataset into the two aforementioned periods (≤1969 and ≥1970) at all scales studied (complete dataset, by altitudinal zones, and by species), because there has been an inflection point in climate data since the early 1970s [48]. Subsequently, we compared the average Julian dates by fitting different models through permutational ANOVAs using the “lmPerm” R package [49], a flexible and very robust analysis that can cope with heteroscedasticity and a wide variety of statistical distributions.

## 3. Results

### 3.1. Phenological Trends at Massif Scale

**Figure 1:**
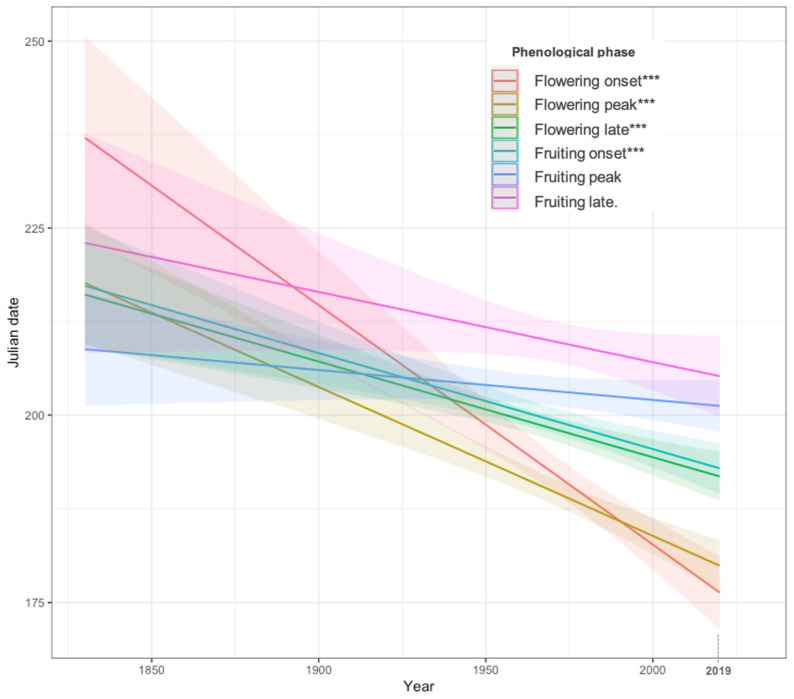
Regression plot showing phenological trends of flowering and fruiting phases over time for different phenophases (from flowering onset to late fruiting) for the assessed period (*p*-value: <2 × 10^−16^ ***; Pseudo-R2: 0.06572040). Lines show negative linear trends for most of the phenophases during the assessed period (1837–2019). The shaded area shows the standard error of the mean.

We found an evident advancement in phenology across the Sierra Nevada massif, as indicated by a significant negative relation between the collection year and the Julian Date of collection applied to entire dataset, regardless of the species (pseudo-R^2^= 0.06572040; *p*-value < 0.001). Further analysis, accounting for the different phenological phases, revealed a consistent trend of advancement in all phases, except for the fruiting peak (non-significant) and fruiting late (marginally significant; Table 1 and Figure 1) phases. Flowering onset exhibited the most pronounced advancement, followed by the flowering peak. Comparing the two periods considered (≤1969 vs. ≥1970), on average, the day of flowering onset shifted from 199 for the 1837–1969 period (*n* = 132) to 188 for the 1970–2019 (*n* = 412) period, indicating an advancement of approximately 11 days. Meanwhile, for the flowering peak phase, an advancement of 13 days was identified for the same period (Table 2). 

### 3.2. Phenological Trends by Altitudinal Range

**Figure 2:**
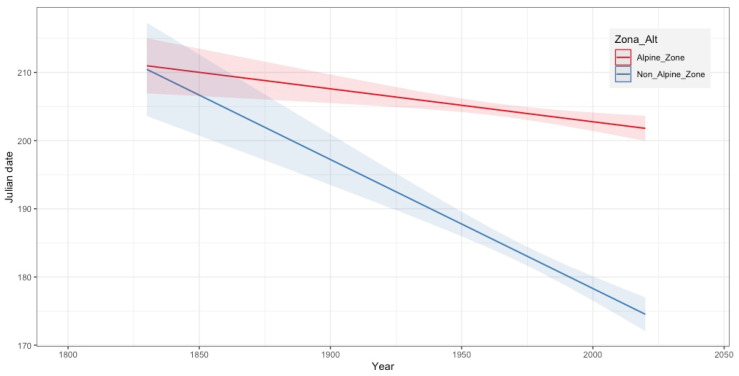
Regression plot showing the linear relationship between Julian date and collection year in each altitudinal zone (alpine vs. non alpine). Note that the trends for both altitudinal zones were negative, yet more pronounced for the non-alpine zone (pseudo-R^2^ = 0.105) than for the alpine zone (pseudo-R^2^ = 0.044).

The fitted models showed a consistent pattern of phenological advancements in the two altitudinal zones considered (alpine vs. non-alpine; pseudo-R^2^ = 0.2782160) for the whole dataset. Remarkably, the phenological advancement (Figure 2) was sharper in the non-alpine zone (pseudo-R^2^ = 0.105) compared to the alpine zone (pseudo-R^2^ = 0.044).

When considering the phenological phases (excluding fruiting peak and fruiting late, where the sample sizes were too small), in both zones, the most significant phenological changes between the two periods considered (pre-1969 and post-1970) were observed in the flowering onset phase (Table 3). Specifically, in the non-alpine zone, the flowering onset shifted from day 194 on average during the period of 1837–1969 (*n* = 30) to day 172 on average for the period of 1970–2019 (*n* = 170), which represents an advancement of approximately 22 days. In the alpine zone, flowering onset occurred, on average, on day 202 during the period of 1837–1969 (*n* = 99) and on day 199 during the period of 1970–2019 (*n* = 237), representing an advancement of 3 days. As for the flowering peak phase in the non-alpine zone and the alpine zone, advancements of 18 and 5 days, respectively, were recorded (Appendix C).

### 3.3. Phenological Trends by Species

The analysis of phenological changes by species when contrasting the two defined periods (pre-1969 and post-1970) showed a significant (or marginally significant) advancement in the flowering peak for eight taxa (Table 4), with the average number of days of advancement varying between 12 (*Lepidium stylatum* Lag. and Rodr.) and 27 (*Ranunculus angustifolius* subsp. *alismoides* (Bory) Malag.). Only in one taxon, i.e., *Scorzoneroides microcephala* (Boiss.) Holub, was the flowering peak significantly delayed, specifically by 20 days, in the post-1970 period. 

Regarding the fruiting peak phase (Table 5), the signal was weaker, and only 4 taxa out of the 12 evaluated showed significant changes when comparing the pre-1969 and post-1970 periods. On the contrary, for two taxa, the fruiting peak was advanced (*Biscutella glacialis* (Boiss. and Reut.) Jord. and *Ranunculus acetosellifolius* Boiss.), and for two others, it was delayed (*Scorzoneroides microcephala* (Boiss.) Holub and *Ranunculus angustifolius* subsp. *alismoides* (Bory) Malag.) 

## 4. Discussion

Our study revealed a generalized advancement in the flowering periods of endemic Sierra Nevada plants, and this trend was consistent both throughout the entire massif and for the two altitudinal ranges analysed. Furthermore, at the species level, the trend pointed in the same direction, although this advance was not significant in all cases. Thus, for Sierra Nevada as a whole, we found that flowering begins, on average, 11 days earlier in the current decade than before the 1970s, which represents an average advancement of 2.2 days/decade. This phenological trend agrees with previous evidence obtained for temperate areas [7,50]. The results were also in line with those identified in the Mediterranean area [20,51], although in some of the studies, the changes were less pronounced [52,53].

One of the novelties of our study is that it focuses on endemic plants, mainly herbs (both annual and perennials) and small shrubs [45], whereas most of the previous phenological studies have analysed mainly trees or large shrubs with wide distribution ranges [20,51,52]. Although the timing of phenological events is driven by complex interactions between living organisms and environmental factors [54,55], climatic variables are particular determinants. Numerous studies have evidenced that consistent phenological advancements in recent decades, not only for plants, but also for other groups of organisms, have been driven primarily by increasing temperatures (e.g., [8,53,56]). Therefore, it is expected that these phenological advancement trends will continue to occur as a consequence of global warming.

Certainly, climatic variables such as temperature change significantly with altitude. In this sense, it was expected that there would be differences between the phenological results obtained in the alpine and non-alpine zones. In line with these expectations, we found an earlier onset of flowering and fruiting in recent decades compared to the decades before 1970 at both altitudinal ranges, but the phenology of lowland endemic plants (non-alpine area) advanced more than that of plants in the alpine area. This does not mean that, in alpine zones, the impact of changes on phenology is low, since as altitude increases, the optimal phenological period shortens, and any minor alteration leads to more noticeable effects. Furthermore, it has long been known that phenology is delayed with altitude (e.g., [57]), but climate warming may further reduce altitude-induced phenological change, as highlighted by [58] over the last six decades. This would have serious consequences in terms of the structure and function of mountain ecosystems.

For endemic plants of Sierra Nevada, we identified that the earliest phases, i.e., flowering onset and peak flowering, showed the most marked advancements. It has also been previously highlighted that global warming particularly affects early phenophases, as the influence on late phases is less pronounced or even not significant [59,60]. In fact, we identified this pattern at the three scales studied (entire massif, by altitudinal ranges, and by particular species). An earlier flowering period can generate serious ecological consequences, such as a mismatch between plant phenology pollinators. In this sense, there is a lack of studies that jointly analyse phenological changes across several organisms, but some of them (e.g., [48]) have proven that, in recent decades, insect phenology has experienced a steeper advancement than that for plants, suggesting a progressive decoupling of some plant–insect interactions, such as pollination, herbivory, or seed predation.

Additionally, our study demonstrates the usefulness of herbarium sheets for long-term phenological monitoring in plants, as has already been proven [30,31]. Therefore, it is crucial to continue to supply herbarium collections with recently collected specimens, and to reverse the current sharply declining trend of the collection rate [30]. However, collecting endemic and threatened plants must be limited for obvious legal and conservation reasons; thus, this type of data could be supplemented with data obtained from direct phenological monitoring in the field.

In conclusion, our study provides valuable insights into the plant phenological changes that have been taking place in recent decades. In particular, we confirmed a strong advancement in plant flowering in the context of a Mediterranean mountain, where this topic had barely been addressed previously. Our results were consistent across scales, and they stand out for the long time period (183 years) and the high number of taxa (83) analysed. In addition, most previous studies have focused on phenological changes in widely distributed trees, but our research deals with poorly studied groups: endemic small shrubs and herbs. Therefore, our results are novel and crucial for biodiversity conservation, since our target species were narrow endemic plants, most of them also being threatened. Moreover, these studies are especially relevant when they affect a diversity hotspot such as Sierra Nevada, which stands out within the Mediterranean hotspot [22], and where the consequences of climate warming are expected to be especially severe [37]. Finally, given that the trend toward phenological advancement in recent decades has been confirmed throughout many territories and scales, it is urgent to address phenological changes at the species level, especially in the case of priority species for conservation. Phenological studies by species would become particularly relevant if interactions with pollinators, dispersers, and other ecosystem groups, as well as the consequences on the different stages of the life cycle of plants, were analysed.

## Figures and Tables

**Table 1:** Summary of the general linear model (model = Julian date ~ year * Phenological phase) for dates (from flowering onset to the late fruiting) during the assessed period (1837–2019) according to the different phenological phases, using the whole dataset of species. Significance levels: *** *p* < 0.001.

Phenological Phase		Intercept	Estimated Coefficient (Year)	±SE	*p*-Value	R-sq. adj
Flowering	Onset	822.14222	−0.31971	±0.05225	1.81 × 10^−9^ ***	0.06461
	Peak	581.28464	−0.1987	±0.03029	8.42 × 10^−11^ ***	0.03901
	Late	450.2716	−0.12795	±0.02491	3.24 × 10^−7^ ***	0.02007
Fruiting	Onset	452.16529	−0.12836	±0.02695	2.17 × 10^−6^ ***	0.02059
	Peak	285.75619	−0.04201	±0.02704	0.13	0.002705
	Late	394.65227	−0.09379	±0.05584	0.093780	0.006428

**Table 2:** Summary of the generalized linear model comparing pre-1969 and post-1970 data for each phenological phase, applied to the whole species dataset. *n* = number of observations for each phenophase and period. Mean day = mean day for each phenophase and period. Change = days of advancement (mean ± SE) when comparing the two periods. Significance levels: *** *p* < 0.001.

Phenological Phase	Period	*n*	Mean Day	Change	*p*-Value	R^2^ adj
Flowering onset	≤1969	132	199	−11 ± 1.39	1.81 × 10^−9^ ***	0.06461
	≥1970	412	188			
Flowering peak	≤1969	295	199	−13 ± 0.93	8.42 × 10^−11^ ***	0.03901
	≥1970	767	186			
Flowering late	≤1969	491	202	−5 ± 0.76	3.24 × 10^−7^ ***	0.02007
	≥1970	799	197			
Fruiting onset	≤1969	323	202	−4 ± 0.81	2.17 × 10^−6^ ***	0.02059
	≥1970	758	198			
Fruiting peak	≤1969	236	204	−1 ± 0.94	0.13	0.002705
	≥1970	611	203			
Fruiting late	≤1969	80	210	−1 ± 1.48	0.093780	0.006428
	≥1970	358	209			

**Table 3:** Summary of the generalized linear model comparing data from alpine vs. non-alpine areas for each phenological phase according to altitudinal zone. Last column shows the number of days of advancement or delay (mean ± SE) according to phenological phase, considering the periods of 1837–1969 and 1970–2019 for each phenological phase. See complete table in (Appendix C). Significance levels: * *p* < 0.05; ** *p* < 0.01; *** *p* < 0.001.

Altitudinal Zone	Phenological Phases		Intercept	Estimated Coefficient (Year)	±Std. Error	*p*-Value	R-sq. adj	Days
Alpine Zone (>2400 m)	Flowering	Onset	527.92939	−0.16624	±0.05684	0.00368 **	0.02497	−3 ± 1.51
		Peak	307.82898	−0.05525	±0.02558	0.0312 *	0.00659	−5 ± 0.85
		Late	312.36614	−0.05523	±0.02593	0.0334 *	0.00464	−1 ± 0.78
	Fruiting	Onset	387.65284	−0.09317	±0.03025	0.00214 **	0.01228	−3 ± 0.84
		Peak	204.57463	0.00135	±0.02961	0.854327	5.993 × 10^−5^	+2 ± 0.99
		Late	170.56605	0.02419	±0.06676	0.362	0.0004877	+7 ± 1.75
Non-Alpine Zone (<2400 m)	Flowering	Onset	985.66581	−0.40963	±0.09499	2.55 × 10^−5^ ***	0.08586	−22 ± 2.39
		Peak	940.34585	−0.38911	±0.08866	1.54 × 10^−5^ ***	0.0553	−18 ± 1.98
		Late	495.47307	−0.15866	±0.05931	0.0079 **	0.02416	−1 ± 1.81
	Fruiting	Onset	459.91797	−0.13807	±0.05064	0.00678 **	0.02442	−1 ± 1.82
		Peak	288.22068	−0.04785	±0.05380	0.37459	0.003022	+4 ± 1.91
		Late	354.59352	−0.08099	±0.09089	0.3743	0.005197	−1 ± 2.36

**Table 4:** Summary of the permutational ANOVAs comparing the phenological differences in flowering peak phases between the two assessed periods (≤1969 vs. ≥1970). Mean_day = average day of flowering peak per period (≤1969 vs. ≥1970). Days (adv-del) = Days (mean ± SE) of advancement (negative) or delay (positive), comparing the two periods. Significance levels: * *p* < 0.05; ** *p* < 0.01; *** *p* < 0.001.

	Taxa	Period	*n*	Mean_Day	Days (adv-del)	*p*-Value
1	*Armeria splendens* (Lag. and Rodr.) Webb	≤1969	7	215	−13 ± 3.82	0.08729
		≥1970	21	202		
2	*Biscutella glacialis* (Boiss. and Reut.) Jord.	≤1969	5	202	−13 ± 2.23	0.05053
		≥1970	29	189		
3	*Centranthus nevadensis* Boiss.	≤1969	6	208	−6 ± 3.69	0.6863
		≥1970	6	202		
4	*Erigeron frigidus* Boiss.	≤1969	24	197	4 ± 2.08	0.2319
		≥1970	29	201		
5	*Jasione amethystine* Lag. and Rodr.	≤1969	6	204	−11 ± 5.2	0.1343
		≥1970	7	193		
6	*Lepidium stylatum* Lag. and Rodr.	≤1969	37	209	−12 ± 5.0	0.0076 **
		≥1970	20	197		
7	*Leontodon boryi* DC.	≤1969	10	207	−9 ± 3.51	0.1221
		≥1970	13	198		
8	*Lomelosia pulsatilloides* (Boiss.) Greuter & Burdet	≤1969	9	216	−19 ± 3.55	<2.2 × 10^−16^ ***
		≥1970	10	197		
9	*Nevadensia purpurea* (Lag. and Rodr.) Rivas Mart.	≤1969	5	190	1 ± 2.61	0.2859
		≥1970	17	191		
10	*Pinguicula nevadensis* (H. Lindb.) Casper	≤1969	5	214	−25 ± 1.33	< 2.2 × 10^−16^ ***
		≥1970	37	189		
11	*Potentilla nevadensis* Boiss.	≤1969	8	199	8 ± 4.3	0.1287
		≥1970	8	207		
12	*Ranunculus acetosellifolius* Boiss.	≤1969	25	184	−20 ± 4.59	0.02086 *
		≥1970	41	164		
13	*Ranunculus angustifolius* subsp. *alismoides* (Bory) Malag.	≤1969	10	218	−27 ± 1.84	<2.2 × 10^−16^ ***
		≥1970	30	191		
14	*Reseda complicata* (Bory)	≤1969	7	196	−2 ± 9.18	0.9804
		≥1970	21	194		
15	*Scorzoneroides microcephala* (Boiss.) Holub	≤1969	23	194	20 ± 3.93	6 × 10^−4^ ***
		≥1970	40	214		
16	*Sideritis glacialis* Boiss. subsp. *glacialis*	≤1969	18	201	6 ± 4.2	0.5811
		≥1970	27	207		
17	*Viola crassiuscula* Bory	≤1969	14	199	−4 ± 2.91	0.7843
		≥1970	35	195		

**Table 5:** Summary of the permutational ANOVAs comparing the phenological differences in fruiting peaks between the two assessed periods (≤1969 vs. ≥1970). Mean_day = average day of flowering peak per period (≤1969 vs. ≥1970). Days (adv-del) = Days (mean ± SE) of advancement (negative) or delay (positive), comparing the two periods. Significance levels: * *p* < 0.05; ** *p* < 0.01; *** *p* < 0.001.

	Taxa	Period	*n*	Mean_Day	Days (adv-del)	*p*-Value
1	*Arenaria pungens* subsp. *Pungens* Clemente ex Lag.	≤1969	16	207	11 ± 6.4	0.3043
		≥1970	19	218		
2	*Biscutella glacialis* (Boiss. and Reut.) Jord.	≤1969	8	220	−30 ± 3.33	<2.2 × 10^−16^ ***
		≥1970	33	190		
3	*Erigeron frigidus* Boiss.	≤1969	15	204	6 ± 3.13	0.4082
		≥1970	19	210		
4	*Erodium boissieri* Coss.	≤1969	5	204	−21 ± 9.76	0.1797
		≥1970	19	183		
5	*Eryngium glaciale* Boiss.	≤1969	7	218	−10 ± 16.73	0.3706
		≥1970	7	208		
6	*Leontodon boryi* DC.	≤1969	22	199	4 ± 3.80	0.5402
		≥1970	24	203		
7	*Lepidium stylatum* Lag. and Rodr.	≤1969	9	212	−13 ± 5.93	0.1747
		≥1970	18	199		
8	*Plantago nivalis* Boiss.	≤1969	30	204	−1 ± 2.93	0.623
		≥1970	53	203		
9	*Ranunculus angustifolius* subsp. *alismoides* (Bory) Malag.	≤1969	7	199	12 ± 1.30	<2.2 × 10^−16^ ***
		≥1970	21	187		
10	*Reseda complicata* (Bory)	≤1969	5	219	17 ± 9.5	0.1628
		≥1970	7	236		
11	*Ranunculus acetosellifolius* Boiss.	≤1969	16	170	−14 ± 2.64	0.0056 **
		≥1970	43	184		
12	*Scorzoneroides microcephala* (Boiss.) Holub	≤1969	17	213	21 ± 5.91	0.0184 *
		≥1970	21	234		

## Data Availability

Please request authors.

## Appendix A

List of taxa selected for this study. Taxa: Accepted scientific name (including authors). *n*: number of samples included for the analysis. Distribution range: according Lorite, et al. (2020) [45]. Conservation status: according Lorite, et al. (2020) [45]: CR= Critically endangered. EN = Endangered. VU = Vulnerable. DD = Data deficient. NT = Near threatened. No_th = not threatened, including Least Concern (LC) and No Evaluated (NE).

	**Taxa**	***n***	**Distribution Range**	**Conservation Status**
1	*Ranunculus acetosellifolius* Boiss.	321	endemic	NT
2	*Scorzoneroides microcephala* (Boiss.) Holub	294	endemic	VU
3	*Ranunculus angustifolius* subsp. *alismoides* (Bory) Malag.	284	endemic	NT
4	*Erigeron frigidus* Boiss.	268	endemic	No_th
5	*Plantago nivalis* Boiss.	261	endemic	No_th
6	*Lepidium stylatum* Lag. & Rodr.	220	endemic	No_th
7	*Leontodon boryi* DC.	212	Iberian Peninsula endemic	NT
8	*Armeria splendens* (Lag. & Rodr.) Webb	163	endemic	VU
9	*Biscutella glacialis* (Boiss. & Reut.) Jord.	162	Iberian Peninsula endemic	NT
10	*Sagina saginoides* subsp. *nevadensi*s (Boiss. & Reut.) Greuter & Burdet	160	endemic	No_th
11	*Sideritis glacialis* Boiss. subsp. *glacialis*	128	Iberian Peninsula endemic	No_th
12	*Viola crassiuscula* Bory	128	endemic	NT
13	*Potentilla nevadensis* Boiss.	121	subendemic	No_th
14	*Jasione amethystina* Lag. & Rodr.	112	endemic	No_th
15	*Eryngium glaciale* Boiss.	109	Iberian_North African	No_th
16	*Nevadensia purpurea* (Lag. & Rodr.) Rivas Mart.	97	endemic	VU
17	*Arenaria pungens* subsp. *pungens* Clemente ex Lag.	88	Iberian Peninsula endemic	No_th
18	*Erodium boissieri* Coss.	81	endemic	VU
19	*Draba hispanica* subsp. *laderoi* Rivas Mart., M.E.García & Penas	80	endemic	No_th
20	*Pinguicula nevadensis* (H. Lindb.) Casper	80	endemic	VU
21	*Reseda complicata* Bory	78	endemic	VU
22	*Lomelosia pulsatilloides* (Boiss.) Greuter & Burdet	70	endemic	No_th
23	*Coincya monensis* subsp. *nevadensis* (Willk.) Leadlay	66	subendemic	NT
24	*Rothmaleria granatensis* (DC.) Font Quer	65	Iberian Peninsula endemic	VU
25	*Centranthus nevadensis* Boiss.	64	Iberian_North African	VU
26	*Erysimum baeticum* (Heywood) Polatschek	63	endemic	No_th
27	*Genista versicolor* Boiss.	63	subendemic	No_th
28	*Leucanthemopsis pectinata* (L.) G.López & C.E.Jarvis	62	endemic	No_th
29	*Thlaspi nevadense* Boiss. & Reut.	61	endemic	VU
30	*Scorzoneroides carpetana* subsp. *nevadensis* (Lange) Izuzq.	60	subendemic	No_th
31	*Sarcocapnos speciosa* Boiss.	56	endemic	VU
32	*Androsace vitaliana* subsp. *nevadensis* (Chiarugi) Luceño	54	endemic	VU
33	*Carex camposii* Boiss. & Reut.	54	subendemic	NT
34	*Linaria glacialis* Boiss.	54	endemic	VU
35	*Thymus serpylloides* Bory subsp. *serpylloides*	54	endemic	No_th
36	*Primula elatior* (L.) L. subsp. *lofthousei* (Hesl.-Harr.) W.W. Sm. & H.R. Fletcher	53	Iberian Peninsula endemic	VU
37	*Sempervivum minutum* (Willk.) Pau	53	subendemic	No_th
38	*Arenaria tetraquetra* subsp. *amabilis* (Bory) H.Lindb.	45	endemic	No_th
39	*Linaria aeruginea* subsp. *nevadensis* (Boiss.) Malag.	44	endemic	No_th
40	*Helianthemum pannosum* Boiss.	41	endemic	VU
41	*Pimpinella procumbens* (Boiss.) H.Wolff.	40	endemic	VU
42	*Centaurea pulvinata* (Blanca) Blanca	39	subendemic	VU
43	*Arenaria nevadensis* Boiss. & Reut.	37	endemic	CR
44	*Carduus carlinoides* subsp. *hispanicus* (Kazmi) Franco	35	endemic	NT
45	*Gentiana pneumonanthe* subsp. *depressa* (Boiss.) Malag.	35	endemic	VU
46	*Gentiana sierrae* Briq.	34	subendemic	VU
47	*Nepeta × boissieri* Willk. (= *N. nepetella* subsp. *laciniata × N. granatensis*)	32	endemic	No_th
48	*Erodium astragaloides* Boiss. & Reut.	30	endemic	CR
49	*Erodium rupicola* Boiss.	28	subendemic	VU
50	*Erysimum nevadense* Reut.	25	endemic	No_th
51	*Senecio nevadensis* Boiss. & Reut.	23	endemic	VU
52	*Armeria filicaulis* subsp. *nevadensis* Nieto Fel., Rosselló & Fuertes	22	endemic	VU
53	*Herniaria boissieri* J.Gay	22	subendemic	NT
54	*Verbascum nevadense* Boiss.	21	subendemic	NT
55	*Alyssum nevadense* P.W.Ball & T.R Dudley	19	endemic	VU
56	*Arabis margaritae* Talavera	19	endemic	CR
57	*Artemisia granatensis* Boiss.	18	endemic	No_th
58	*Iberis carnosa* subsp. *embergeri* (Serve) Moreno	17	endemic	EN
59	*Thymus granatensis* subsp. *granatensis* Boiss.	17	Iberian Peninsula endemic	No_th
60	*Campanula rotundifolia* subsp. *willkommii* (Witasek.) Blanca	15	endemic	No_th
61	*Cerastium alpinum* subsp. *nevadense* (Pau) Mart.Parras & Molero Mesa	14	endemic	No_th
62	*Chamaespartium undulatum* (Ern) Talavera & L.Sáez	14	subendemic	VU
63	*Pedicularis verticillata* subsp. *caespitosa* (Webb) I.Soriano	14	endemic	VU
64	*Chaenorhinum glareosum* (Boiss.) Willk.	13	endemic	No_th
65	*Laserpitium latifolium* subsp. *nevadense* Mart.-Lirola, Molero Mesa & Blanca	12	endemic	No_th
66	*Artemisia alba* Turra subsp. *nevadensis* (Willk.) Blanca & Morales	10	Iberian Peninsula endemic	VU
67	*Vaccinium uliginosum* subsp. *nanum* (Boiss.) Rivas Mart., Asensi, Molero Mesa & F.Valle	10	endemic	No_th
68	*Odontites viscosus* subsp. *granatensis* (Boiss.) Bolliger	9	endemic	CR
69	*Taraxacum nevadense* H.Lindb.	9	subendemic	No_th
70	*Alchemilla fontqueri* Rothm.	8	endemic	CR
71	*Cytisus galianoi* Talavera & Gibbs	8	subendemic	NT
72	*Helianthemum appeninum* subsp. *estevei* (Peinado & Mart.Parras) G.López	8	endemic	VU
73	*Moehringia fontqueri* Pau	8	endemic	EN
74	*Nepeta nepetella* subsp. *laciniata* (Willk.) Aedo	8	endemic	No_th
75	*Ranunculus cherubicus* subsp. *girelae.* Fern.-Prieto et al.	8	endemic	DD
76	*Thymus × pseudogranatensis* Vizoso, F.B.Navarro & Lorite (= Th. granatensis subsp. granatensis × Th. zygis subsp. Gracilis	7	endemic	No_th
77	*Laserpitium longiradium* Boiss.	6	endemic	No_th
78	*Hippocrepis nevadensis* (Hrabetová) Talavera & E.Domínguez	5	endemic	VU
79	*Cerastium alpinum* subsp. *aquaticum* (Boiss.) Mart.Parras & Molero Mesa	4	endemic	No_th
80	*Narcissus nevadensis* Pugsley subsp. *nevadensis*	4	subendemic	CR
81	*Pedicularis comosa* subsp. *nevadensis* (Pau) A.M.Romo	4	endemic	VU
82	*Centaurea bombycina* subsp. *xeranthemoides* (Lange) Blanca, Cueto & M.C.Quesada	3	endemic	VU
83	*Cirsium x nevadense* Willk.	3	Iberian Peninsula endemic (hybrid)	No_th
84	*Hippocrepis prostrata* Boiss.	3	endemic	CR
85	*Linaria saturejoides* subsp. *angustealata* (Willmott) Malag.	3	subendemic	No_th
86	*Salix hastata* subsp. *sierrae-nevadae* Rech.f.	3	endemic	CR
87	*Tephroseris elodes* (Boiss.) Holub subsp. *elodes*	3	endemic	EN
88	*Armeria filicaulis* subsp. *trevenqueana* Nieto Fel.	2	endemic	EN
89	*Artemisia × fragosoana* Font Quer (= *A. granatensis × A. umbelliformis*)	2	endemic	No_th

## Appendix B

Number of observations per year. Year: Years with at least one record. Frequency: Number of observations per given year.

**Year**	**Frequency**
1837	5
1851	125
1852	27
1853	2
1858	1
1871	1
1879	1
1891	11
1895	2
1898	5
1901	1
1902	1
1906	1
1907	9
1908	26
1921	21
1923	366
1925	1
1928	1
1929	8
1930	174
1931	1
1933	1
1934	11
1935	99
1941	4
1942	1
1943	1
1944	39
1946	21
1947	59
1950	3
1951	41
1953	79
1954	26
1955	10
1957	3
1958	14
1959	7
1960	8
1963	3
1964	34
1965	4
1966	13
1967	122
1968	100
1969	64
1970	137
1971	127
1972	93
1973	133
1974	85
1975	75
1976	259
1977	59
1978	304
1979	104
1980	296
1981	198
1982	141
1983	223
1984	231
1985	83
1986	72
1987	125
1988	205
1989	62
1990	125
1991	17
1992	20
1993	28
1994	16
1995	29
1996	118
1997	44
1998	51
1999	20
2000	13
2001	3
2002	12
2003	21
2004	4
2006	1
2007	7
2008	3
2009	6
2010	54
2011	24
2012	9
2013	13
2014	30
2015	2
2016	1
2017	18
2019	4

## Appendix C

Number of days in advance or delay for each phenological phase by altitudinal zone according to periods. Period (years): detailed period range that differentiates those species data found before 1969 and after 1970. n: number of observations for all phenological phases. Mean_day: average day between periods; n_days: number of days advanced (−) or delayed (+) and (±SE) standard deviation of advanced and delay days. Trends: “advance” if is negative the number of days and “delayed” if is positive the number of days.

**Altitudinal Zone**	**Phenological Phase**	**Period (Years)**	**(n)**	**Mean_Day**	**n_Days**	**Trends**
No Alpine (<2400 m)	Flowering early	≤1969	30	194	−22 ± 2.39	Advanced
		≥1970	170	172		
	Flowering peak	≤1969	48	185	−18 ± 1.98	Advanced
		≥1970	283	167		
	Flowering late	≤1969	70	182	−1 ± 1.81	Advanced
		≥1970	223	181		
	Fruiting early	≤1969	65	187	−1 ± 1.82	Advanced
		≥1970	236	186		
	Fruiting peak	≤1969	50	191	4 ± 1.91	Delayed
		≥1970	213	195		
	Fruiting late	≤1969	14	195	−1 ± 2.36	Advanced
		≥1970	140	194		
Alpine (>2400 m)	Flowering early	≤1969	99	202	−3 ± 1.51	Advanced
		≥1970	237	199		
	Flowering peak	≤1969	236	202	−5 ± 0.85	Advanced
		≥1970	469	197		
	Flowering late	≤1969	412	204	−1 ± 0.78	Advanced
		≥1970	562	203		
	Fruiting early	≤1969	250	206	−3 ± 0.84	Advanced
		≥1970	513	203		
	Fruiting peak	≤1969	178	206	2 ± 0.99	Delayed
		≥1970	387	208		
	Fruiting late	≤1969	59	212	7 ± 1.75	Delayed
		≥1970	212	219		
